# Genetic insights of blood lipid metabolites on polycystic ovary syndrome risk: a bidirectional two-sample Mendelian randomization study

**DOI:** 10.3389/fendo.2024.1391826

**Published:** 2024-07-09

**Authors:** Xinzhe Wang, Huawei Han, Xiuwen Shi, Xiaping Nie, Rui Zhu, Jing Jin, Huifang Zhou

**Affiliations:** ^1^ Department of Gynecology, Affiliated Hospital of Nanjing University of Chinese Medicine, Nanjing, China; ^2^ Department of Orthopedics, The Second Affiliated Hospital of Nanjing University of Chinese Medicine, Nanjing, China

**Keywords:** Mendelian randomization, lipid metabolites, polycystic ovarian syndrome, genetics, meta-analysis, metabolic pathway analysis

## Abstract

**Background:**

Pathologically, metabolic disorder plays a crucial role in polycystic ovarian syndrome (PCOS). However, there is no conclusive evidence lipid metabolite levels to PCOS risk.

**Methods:**

In this study, genome-wide association study (GWAS) genetic data for 122 lipid metabolites were used to assign instrumental variables (IVs). PCOS GWAS were derived from a large-scale meta-analysis of 10,074 PCOS cases and 103,164 controls. An inverse variance weighted (IVW) analysis was the primary methodology used for Mendelian randomization (MR). For sensitivity analyses, Cochran Q test, MR-Egger intercept, MR-PRESSO, leave-one-out analysis,and Steiger test were performed. Furthermore, we conducted replication analysis, meta-analysis, and metabolic pathway analysis. Lastly, reverse MR analysis was used to determine whether the onset of PCOS affected lipid metabolites.

**Results:**

This study detected the blood lipid metabolites and potential metabolic pathways that have a genetic association with PCOS onset. After IVW, sensitivity analyses, replication and meta-analysis, two pathogenic lipid metabolites of PCOS were finally identified: Hexadecanedioate (OR=1.85,95%CI=1.27–2.70, *P*=0.001) and Dihomo-linolenate (OR=2.45,95%CI=1.30–4.59, *P*=0.005). Besides, It was found that PCOS may be mediated by unsaturated fatty acid biosynthesis and primary bile acid biosynthesis metabolic pathways. Reverse MR analysis showed the causal association between PCOS and 2-tetradecenoyl carnitine at the genetic level (OR=1.025, 95% CI=1.003–1.048, *P*=0.026).

**Conclusion:**

Genetic evidence suggests a causal relationship between hexadecanedioate and dihomo-linolenate and the risk of PCOS. These compounds could potentially serve as metabolic biomarkers for screening PCOS and selecting drug targets. The identification of these metabolic pathways is valuable in guiding the exploration of the pathological mechanisms of PCOS, although further studies are necessary for confirmation.

## 1 Introduction

Globally, PCOS affects 2.2% to 26% of women, making it the most prevalent reproductive and endocrine disorder (1). The clinical manifestations are varied, such as menstrual disorders, infertility, hirsutism, acne, glucose and lipid metabolism disorder (2). Moreover, it can increase the risk of cardiovascular disease, diabetes, gynecological tumors and other diseases (3, 4). PCOS is a multisystem disease that affects women from adolescence to menopause, imposing a heavy economic burden on patients and posing threats to their physical and mental well-being. The delayed detection and inadequate management of PCOS have generated discontent among women worldwide (5). Therefore, exploring the key targets for the pathogenesis and treatment of PCOS has increasingly become a public health issue that requires urgent attention.

Metabolites, which are the outcomes of genetic and environmental influences on organisms, exhibit sensitivity to physiological and pathological variations within the body (6). Metabolomics play an imperative role identifying pertinent biomarkers for disease diagnosis and treatment. Numerous clinical and basic studies in recent years have reported the effects of lipid metabolism on PCOS. Lipid metabolism disorders and PCOS can form a detrimental cycle, which can be responsible for the main pathological features of PCOS, including insulin resistance and hyperandrogenism (7).Moreover, certain lipid metabolites’ aberrant changes are implicated in the chronic low-grade inflammation process of PCOS, consequently influencing oocyte maturation and exacerbating ovulation disorders (8, 9). Yu et al. (10) reported that the blood lipid metabolites of PCOS differed significantly from those of healthy controls. They also observed alterations in related pathway profiles, such as fatty acid degradation and ether lipid metabolism. Additional, Buszewska-Forajta et al. (11) found a deterioration of lipid metabolism in PCOS patients, with higher sphingolipids and lower fatty acids. These studies are, however, mostly cross-sectional or observational, rendering their conclusions susceptible to confounding factors. Currently, there is no detailed and in-depth study exploring whether blood lipid metabolites are causally associated with PCOS.

Genetic causality can be determined by Mendelian randomization (MR), an emerging and effective scientific technique. The genetic variants or single nucleotide polymorphism (SNPs) may be used as an useful instrument to assess causal link between exposure and outcome (12). By circumventing reverse causality and confounding factors encountered in traditional epidemiological studies, MR can provide scientifically reliable results for elucidating disease pathogenesis (13). Therefore, it has gained widespread adoption for investigating PCOS pathology (14). In this study, we utilized extensive GWAS data to explore the genetic relationship between blood lipid metabolites and PCOS through bidirectional two-sample MR. Our findings aim to provide innovative insights directed towards the prevention and treatment of PCOS.

## 2 Methods

### 2.1 Study design

**Figure 1:**
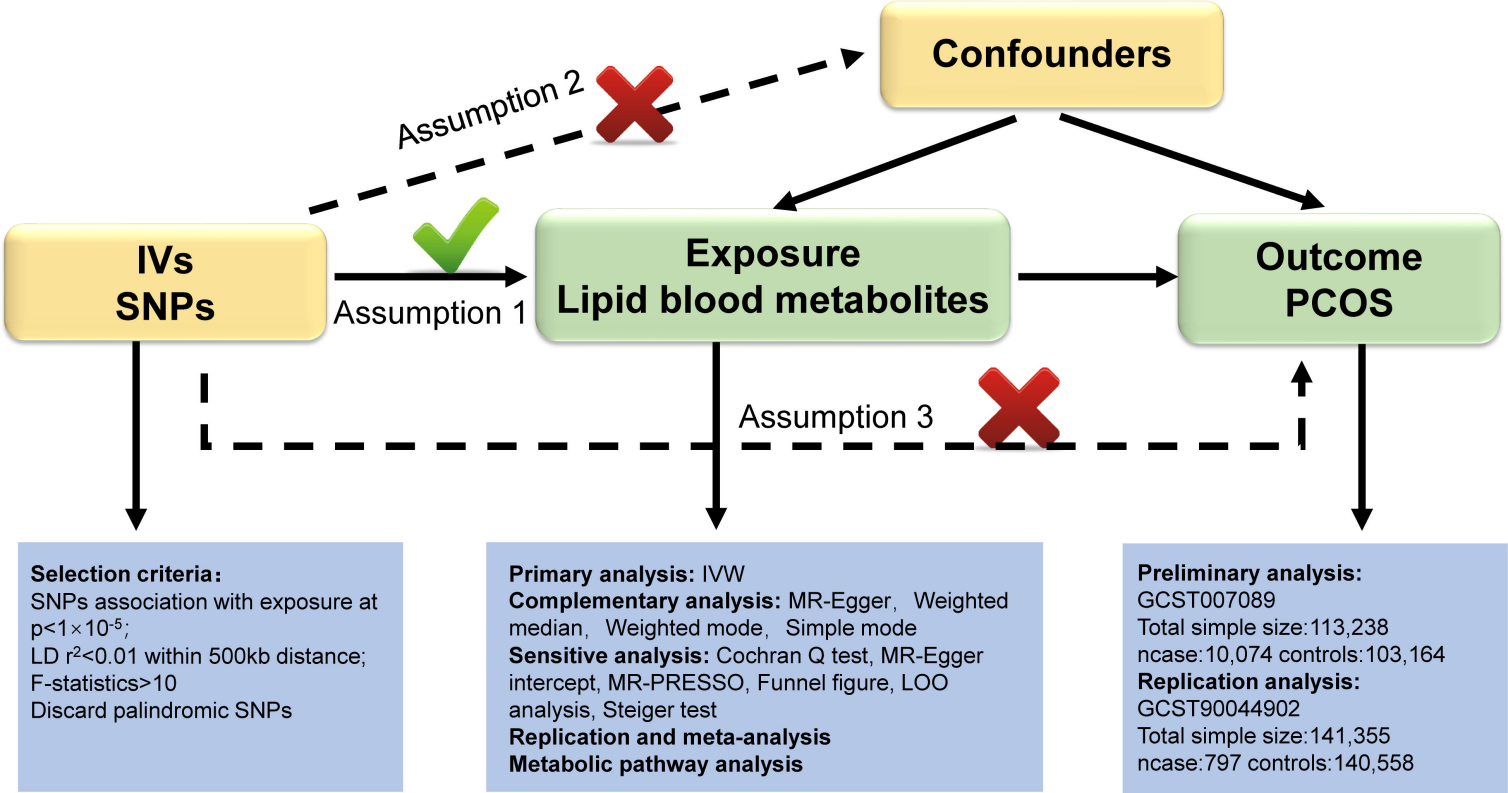
Overview of this Mendelian randomization (MR) analysis. Abbreviations: IV, instrumental variables; SNPs, single nucleotide polymorphisms; IVW, inverse variance weighted; LD, linkage disequilibrium; LOO analysis, leave- one- out analysis; MR- PRESSO, MR- Pleiotropy Residual sum and outlier.

In our study, we utilized validated genetic variants from publicly available GWAS data as instrumental variables (IVs) to investigate their causal relationship with outcome by replacing exposure. Blood lipid metabolites were preliminarily considered as “exposure”, while PCOS was considered the “outcome”. Our study design adhered to the three fundamental assumptions of MR outlined by Bowden et al. (15): (1) The IVs should exhibit a strong connection with the blood lipid metabolites; (2) IVs should not be influenced by any confounding factors between blood lipid metabolites and PCOS; (3) IVs must solely impact PCOS through blood lipid metabolites. These assumptions ensure that the genetic variants randomly assigned during meiosis are strongly linked to lipid metabolites, unaffected by confounding factors or reverse causality, thereby ensuring the validity of causal inferences. **Figure 1** illustrates the three hypotheses and the research methodologies employed. We established stringent criteria for IV selection to fulfill hypothesis 1. Primary and supplementary analyses in MR were used to systematically assess the causal effects of lipid metabolites on PCOS. A series of sensitivity analyses were performed to confirm that hypotheses 2 and 3 were not violated. Following preliminary analysis, we utilized another PCOS GWAS dataset (Number: GCST90044902) to conduct replication and meta-analysis for further screening of target lipid metabolites. Metabolic pathway analysis and reverse analysis were also performed to make findings.

### 2.2 GWAS data for 122 blood lipid metabolites and PCOS

Blood lipid metabolite GWAS data were identified by Shin et al. using genome-wide association scans and high-throughput metabolic measurements. They assessed the genetic relevance of 122 lipid metabolites in 7824 individuals of European ancestry using nearly half a million SNPs (16). Access to the metabolomics GWAS server was made (http://metabolomics.helmholtz-muenchen.de/gwas).

PCOS GWAS stem from a comprehensive meta-analysis conducted by Day et al. that utilized Rotterdam criteria, National Institutes of Health (NIH) criteria, or self-reported criteria to diagnose PCOS. 10,074 patients and 103,164 controls of European descent were enrolled in this study (17). The data accessing code is GCST007089 and access is available via the GWAS catalog (https://www.ebi.ac.uk/gwas).

It is important to highlight that weak instrument bias resulting from sample overlap can influence the observational association between exposure and outcome, potentially leading to inflated type I error rates for causal effects (18). In our study, we selected samples from different datasets to mitigate the bias caused by overlapping samples.

### 2.3 Instrumental variables selection

A rigorous set of criteria was set to screen for excellent IVs related to exposure. 1) We extracted IVs with significance thresholds below 1×10^−5^ in GWAS data of exposure, which are considered to be highly correlated with exposure. Our linkage disequilibrium (LD) was set at 0.01 within 500 KB to ensure independence of IVs.2) F-statistic was used to quantify the strength of genetic variation for individual SNP. IVs with F-statistic <10 were removed to reduce the possibility of weak instrument bias. F = (n-k-1) R^2^/k (1-R^2^) (n represents the sample size, k represents the number of IVs included, and R^2^ represents the instrumental variable explaining the degree of exposure) (19).3) IVs were extracted from outcome GWAS data, and the alleles of exposure- and outcome-SNPs were harmonized. We then eliminated palindromic SNPs with intermediate effect allele frequencies (EAF > 0.42).

### 2.4 Primary analysis and sensitivity analysis

When all included SNPs are valid instrumental variables, the IVW method can provide accurate and unbiased estimates of causality (20).So IVW method was considered as the primary method to evaluate the causal relationship between lipid metabolites and PCOS (*P <*0.05 was considered significant). Additionally, we employed the weighted median (WM), MR-Egger, simple mode, and weighted mode methods as supplementary analyses. The WM grants robust outcomes for causal analyses when less than half of the SNPs are deemed invalid (21). MR-Egger can access the horizontal pleiotropy of all invalid IVs (22). Weighted mode and simple mode were also employed to address bias resulting from a limited number of IVs that do not meet MR’s causal inference criteria (23). Significance estimates provided by the primary analysis were considered to be significant if they were in the same direction as those provided by the supplementary analysis. Five methods are helpful to achieve the comprehensive evaluation of causal effects.

A series of sensitivity analyses were conducted to verify that the findings did not violate the core MR assumptions and to enhance the reliability of causal effects. Cochran’s Q statistic was conducted to quantify the heterogeneity among IVs. The egger-intercept method can assess whether instrumental variables are related to confounding factors and test whether causal effects are biased by horizontal pleiotropy (20, 24) (*P >*0.05 indicated no significant heterogeneity and horizontal pleiotropy). Furthermore, the MR-PRESSO method was utilized to identify and correct for any significant outliers and to address the limited statistical power of the Egger-intercept method in order to reduce pleiotropic bias (25). Leave-one-out sensitivity analysis involved systematically excluding individual SNPs one by one to assess the impact on effect estimates and to ensure that the causal inference was not heavily reliant on a single SNP, thus ensuring the robustness of the overall causal conclusions. Finally, We also performed the Steiger method to verify that the selected SNPs explained greater variability in the exposure than in the outcome(*P <*0.05), which is important to avoid reverse causality bias in the study (26).

### 2.5 Replication and meta-analysis

To further validate the identification of lipid metabolites based on the mentioned criteria, we conducted a repeat IVW analysis using data from another GWAS in the GWAS catalog involving 141,355 individuals of European ancestry (GCST90044902) (27). The results from both the initial and replication analyses were then combined in a meta-analysis to strengthen the evidence supporting the genetic association of protective and pathogenic lipid metabolites with PCOS.

### 2.6 Metabolic pathway analysis

The KEGG database metabolic pathway analysis was conducted using MetaboAnalyst 5.0 (https://www.metaboanalyste.ca/) (28) to explore the potential pathogenesis of lipid metabolites affecting the pathology of PCOS. *P*<0.1 was considered statistically significant (29).

### 2.7 Reverse MR analysis

In order to in-depth and comprehensive analysis of blood lipid metabolites and PCOS genetic causality, we regarded PCOS as “exposed”, and the identified blood lipid metabolites as “outcome”. Similarly, we set the significance threshold at 1×10^−5^ and LD r^2^<0.01 within 500KB to screen for IVs strongly associated with PCOS. Palindromic SNPs were removed and weak IVs interference was excluded. Then, we applied IVW methods to investigate whether the PCOS onset contributes to genetic changes in lipid metabolites. Results were robustly tested using sensitivity analysis.

## 3 Results

### 3.1 IVs selection

Following meticulous selection of SNPs strongly associated with every lipid metabolite, SNPs for caprate (10:0) and butyrylcarnitine were not found in harmonizing with the PCOS GWAS. Consequently, MR of these two metabolites were abandoned. Details of the corresponding SNPs for the remaining 120 lipid metabolites for MR analysis are provided in **Supplementary Table S2**. These SNPs contains no palindrome SNPs and F statistic > 10, be deemed to be valid IVs.

### 3.2 Primary analysis and sensitivity analysis

**Figure 2:**
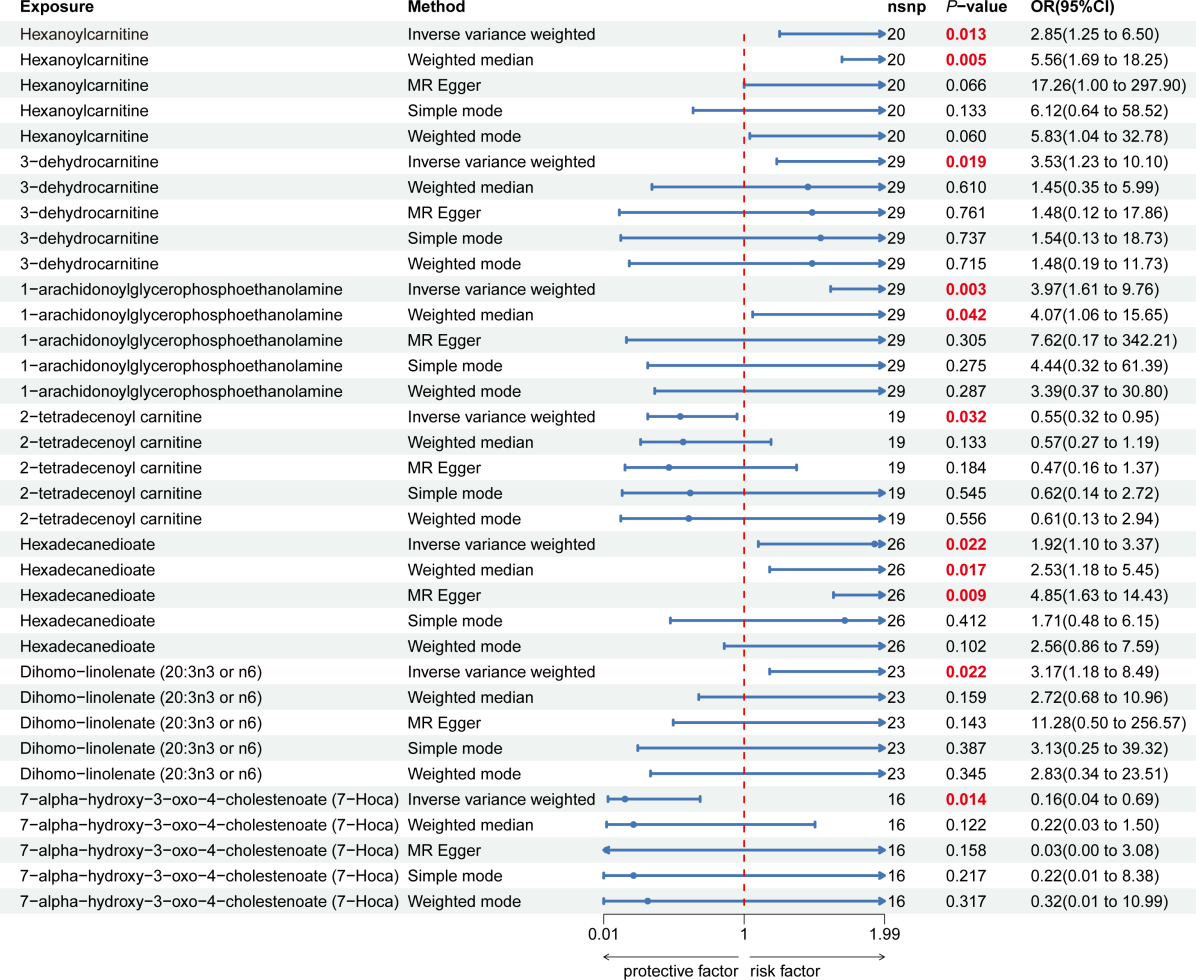
Forest plot of the causal relationship between 7 lipid metabolites and PCOS in the result of primary and supplementary analyses The blue dots represent the estimates of five methods, and the blue bars represent the 95% confidence intervals of estimates. The OR > 1 indicates increased risk while< 1 indicates decreased risk.

**Figure 3:**
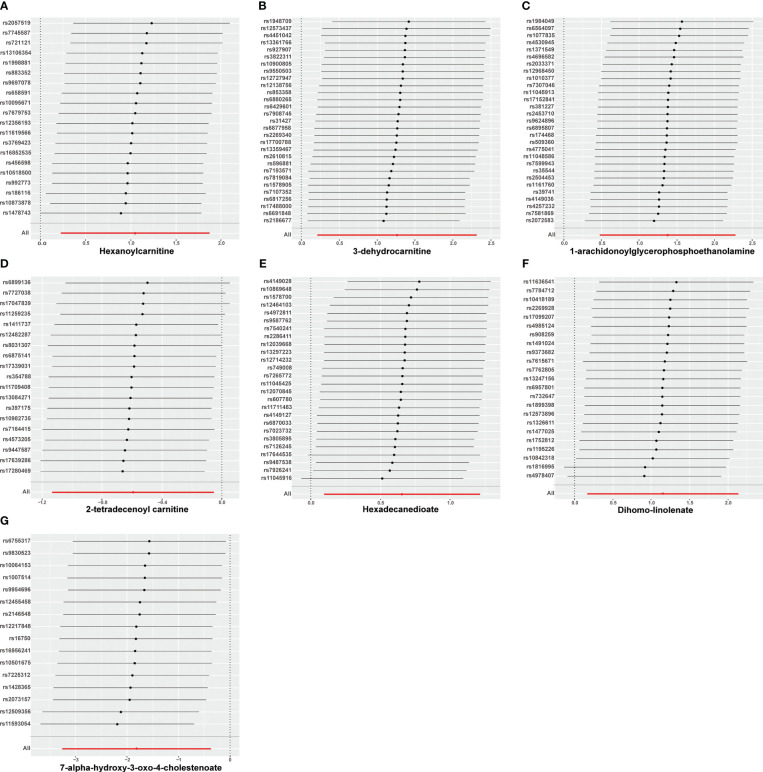
Leave-one-out analysis of causal estimates of exposure (Specific lipid metabolites) on polycystic ovarian syndrome(PCOS). Calculate the MR results of the remaining IVs after removing the IVs one by one. **(A)**:*Hexanoylcamitine;* **(B)**: *3-dehydrocarnitine*; **(C)**: *1-arachidonoylglycerophosphoethanolamine*; **(D)**: *2-tetradecenoyl carnitine*; **(E)**: *Hexadecanedioate*; **(F)**: *Dihomo-linolenate*; **(G)**: *7-alpha-hydroxy-3-oxo-4-cholestenoate*;.

Nine lipid metabolites were preliminarily identified to be associated with PCOS using the IVW method (IVW P<0.05) (**Supplementary Table S3**). The direction of the odds ratio (OR>1 or OR<1) indicates the positive or negative correlation trend of the causal effect. Further analysis revealed inconsistent OR values among the five methods, leading us to conclude that the causal relationship between dehydroisoandrosterone sulfate (DHEA-S), 2-linoleoylglycerophosphocholine, and PCOS may be a false positive. Therefore, after preliminary analysis, we identified 2-tetradecenoyl carnitine(IVW OR=0.55, 95% CI=0.32–0.95, *P*=0.032) and 7-alpha-hydroxy-3-oxo-4-cholestenoate (7-Hoca) (IVW OR=0.16, 95% CI=0.04–0.69, *P*=0.014)had causal relationship with the decreased risk of PCOS. While the remaining 5 metabolites had causal relationship with the increased risk of PCOS: hexanoylcarnitine(IVW OR=2.85,95%CI=1.25–6.50,*P*=0.013), 3-dehydrocarnitine(IVW OR=3.53,95%CI=1.23–10.10,*P*=0.019), 1-arachidonoylglycerophosphoethanolamine(IVW OR=3.97,95%CI=1.61–9.76,*P*=0.033), hexadecanedioate(IVW OR=1.92, 95% CI=1.10–3.37, *P*=0.022) and dihomo-linolenate (20:3n3 or n6) (IVW OR=3.17, 95% CI=1.18- 8.49, *P*=0.022) (**Figure 2**). Scatter plots show the MR effects of the seven metabolites estimated by the different methods with PCOS (**Supplementary Figure S1**). The results of a series of sensitivity analyses ensured the robustness of causal effects. These seven metabolites did not appear to be linked to PCOS by horizontal pleiotropy or heterogeneity according to Cochran’s Q statistics and MR-Egger (all *P*>0.05) (**Table 1**). The results of MR-PRESSO did not support the presence of outlying SNPs (all *P*>0.05) (**Table 1**). The distribution of SNPs for each metabolite is presented using funnel plots. IVW showed that SNPs were distributed roughly symmetrically, indicating that the results of the MR analysis were not biased by outlying SNPs (**Supplementary Figure S2**). The plot of leave-one-out analysis showed that when one snp was removed, the overall causal effect of the remaining SNPs on the outcome did not deviate substantially, supporting that individual SNPs were not responsible for biasing estimates of the MR total effects (**Figure 3**). The Steiger test revealed that the results of lipid metabolites and PCOS causality were not interfered by reverse causality effect (all *P*<0.05) (**Supplementary Table S4**).

**Table 1:** The results of heterogeneity, horizontal pleiotropy and MR-PRESSO of the 7 lipid metabolites and PCOS in the MR analysis.

Exposure	Outcome	Pleiotropy test		Heterogeneity test		MR-PRESSO	
		Intercept	p-value	Q	Q_ p-value	Sd	Global Test p-value
Hexanoylcarnitine	PCOS	-0.04	0.21	15.82	0.67	0.384	0.72
3-dehydrocarnitine	PCOS	0.02	0.46	33.80	0.21	0.536	0.23
1-arachidonoylglycerophosphoethanolamine	PCOS	-0.01	0.73	28.20	0.45	0.460	0.50
2-tetradecenoyl carnitine	PCOS	0.01	0.73	12.94	0.79	0.234	0.82
Hexadecanedioate	PCOS	0.02	0.07	31.37	0.18	0.286	0.18
Dihomo-linolenate (20:3n3 or n6)	PCOS	-0.02	0.41	21.50	0.49	0.497	0.47
7-alpha-hydroxy-3-oxo-4-cholestenoate (7-Hoca)	PCOS	0.03	0.45	11.69	0.70	0.650	0.67

MR, Mendelian randomization; Q, heterogeneity statistic Q.

### 3.3 Replication and meta-analysis

**Figure 4:**
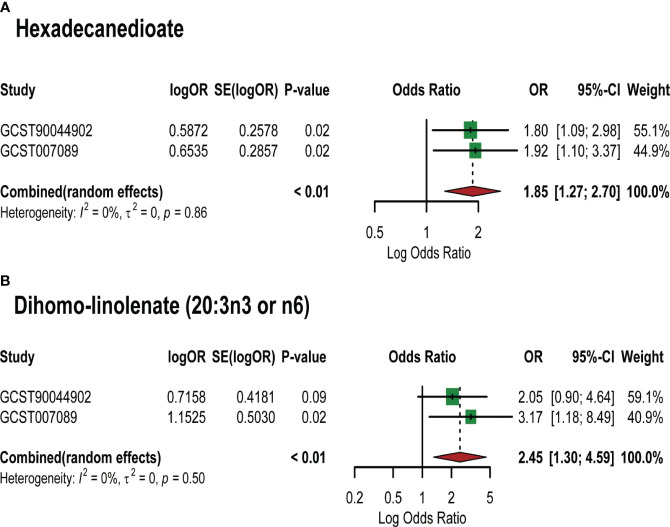
Meta-analysis of the causal associations between lipid metabolites and PCOS. GCST007089: GWAS for PCOS used in the primary analysis; GCST90044902: GWAS for PCOS used in the replication analysis. **(A)** Meta-analysis results of MR analysis of hexadecanedioate and different PCOS GWAS; **(B)** Meta-analysis results of MR analysis of dihomo-linolenate (20:3n3 or n6) and different PCOS GWAS. Abbreviations: SE: Standard Error; 95%- CI: 95% confidence interval; OR: odds ratio.

Based on the PCOS GWAS data from the FinnGen database, we used 7 identified lipid metabolites as “exposures” to conduct replication analysis and meta-analysis of their causal relationship with PCOS. Common effect model and random effect model ultimately confirmed a strong causal association between two lipid metabolites and PCOS (**Supplementary Table S5**). Specifically, Hexadecanedioate (OR=1.85,95%CI=1.27–2.70, *P*=0.001) and dihomo-linolenate (20:3n3 or n6) (OR=2.45,95%CI=1.30–4.59, *P*=0.005) were found to be pathogenic lipid metabolites in PCOS (**Figure 4**). However, the meta-analysis did not reveal a significant causal relationship between the remaining five metabolites and PCOS onset, such as 3-dehydrocarnitine and 2-tetradecenoyl carnitine (*P* value>0.05). This may be explained by the limited sample size of the PCOS GWAS data used in the replication analysis and the difference in the two PCOS GWAS samples.

### 3.4 Metabolic pathway analysis

The selected 7 lipid metabolites were used to explore their possible metabolic pathways involved in PCOS pathology. Despite not identifying any metabolic pathways at the 0.05 significance level, the researcher adjusted the significance threshold to P<0.1 to consider findings as statistically significant (29). The results highlighted two significant metabolic pathways: biosynthesis of unsaturated fatty acids (*P*=0.068) and primary bile acid biosynthesis (*P*=0.086), which are believed to contribute to the development and progression of PCOS.

### 3.5 Reverse MR analysis

According to the reverse MR analysis, we identified the genetic association between PCOS and 2-tetradecenoyl carnitine (IVW OR=1.025, 95% CI=1.003–1.048, *P*=0.026) (**Supplementary Table S6**). Heterogeneity and horizontal pleiotropy were not significant in the sensitivity analysis (*P*>0.05).

## 4 Discussion

The polycystic ovary syndrome is an endocrine and metabolic disorder with genetic heterogeneity that affects reproductive health (30).Metabolic disorders, particularly lipid metabolism disorders, are prominent clinical manifestations and key mechanisms of PCOS (31). Recent research has highlighted the significance of identifying differential blood metabolites in understanding the pathogenesis of PCOS (32). However, the specific blood lipid metabolites that may have protective or pathogenic effects on the development of PCOS remain unclear. GWAS from publicly available sources were used in this study to provide the first comprehensive and in-depth exploration of the gene-level link between blood lipid metabolites and PCOS. Through IVW and sensitivity analysis, we identified 7 lipid metabolites that are causally relate to PCOS. Subsequent replication and meta-analysis further confirmed the link between hexadecanedioate, dihomo-linolenate and the increased risk of PCOS, ensuring the robustness of the findings. Additionally, we identified two metabolic pathways that may contribute to PCOS’ biological mechanisms. These findings provide ideas for diagnosing and treating PCOS with specific lipid metabolites.

Hexadecanedioate, a long-chain dicarboxylic acid, is synthesized via the ω-oxidation pathway (33). Raji’s early studies linked hexadecanedioate to increased all-cause mortality in women and highlighted its negative impact on women’s health (34). However, there is a current lack of studies investigating the role of hexadecanedioate in the development of PCOS. Hexadecanedioate is recognized as a natural substrate for the organic anion-transporting polypeptide (OATP) (35). Francisca et al. (36) observed elevated OATP levels in women with PCOS compared to those without. OATP regulates the uptake of dehydroepiandrosterone sulfate (37),which may be involved in the metabolic process of hyperandrogenism in PCOS. This provides a possible explanation for the mechanism of hexadecanedioate as a pathogenic lipid metabolite in PCOS. Among the 26 SNPs identified as instrumental variables for hexadecanedioate, rs11045916(β=0.0605, SE=0.0069, *P*=1.424E-18) (**Supplementary Table S2**) (**Supplementary Figure S3**) showed the strongest association. However, the specific role of this genetic variant in PCOS pathogenesis remains unclear.

Zhang et al. (38) reported an increase in dihomo-linolenate levels in plasma phospholipids in individuals with PCOS. Our study also confirmed the relationship between dihomo-linolenate and PCOS, but the exact mechanism by which dihomo-linolenate contributes to PCOS pathogenesis is still unknown. In a recent review, dihomo-linolenate and its derivatives were discussed as possible mediators of inflammation (39). It can be converted to arachidonic acid, which exhibits proinflammatory properties and may contribute to the chronic inflammatory state observed in PCOS. A cross-sectional study revealed that higher linolenic acid was link to glucose and lipid metabolism disorders, as well as increased insulin resistance (40). However, more research is necessary to determine if linolenic acid directly increases the risk of PCOS by aggravating insulin resistance. Additionally, in the dihomo-linolenate GWAS data, we identified a causal relationship between rs4978407, rs10842318, rs1816995, and PCOS (**Supplementary Figure S3**). These genetic loci should be further investigated as potential key factors in the pathogenesis of PCOS. Inverse MR analysis revealed interesting results, indicating a decrease in 2-tetradecenoyl carnitine levels with the onset of PCOS. Despite the negative results in the meta-analysis for 2-tetradecenoyl carnitine, this lipid metabolite remains significant. The statistical significance of bidirectional causal studies suggests that it could be a promising target for PCOS treatment.

In the pathways analysis, it has been reported that the biosynthesis of unsaturated fatty acids is crucial to the metabolic pathway of lipid metabolites that affect the risk of PCOS. Polyunsaturated fatty acid metabolism has been found to improve sex hormone disorders, reduce oxidative stress, and reduce inflammation in PCOS, according to a meta-analysis (41). Ma et al.’s animal experiment confirmed that polyunsaturated fatty acids can enhance oocyte quality in PCOS mice by reducing oxidative stress level and improving spindle abnormalities (42). The lipid metabolites identified in our study may serve as important targets in the biosynthetic pathway of unsaturated fatty acids that affect PCOS. We also found that the primary bile acid biosynthesis pathway is of substantial significance. Bile acids contribute greatly to cholesterol metabolism and are known to be important endocrine regulators (43). This pathway has been associated with metabolic diseases such as diabetes mellitus and non-alcoholic fatty liver disease (44, 45). Yu et al. (46) investigated the bile acid profiles in PCOS and controls, and discovered that the bile acid anabolic pathways were crucial for glucose metabolism disorder in PCOS. The next phase of research in this field should focus on the exploration of the downstream molecules in this pathway and the underlying mechanisms involved in PCOS pathology.

Our study has certain advantages. Firstly, we utilized the most comprehensive published GWAS, covering large populations to ensure the objectivity of our results. Secondly, after strict screening effective IVs, rational MR and sensitivity analyses were used to thoroughly evaluate the causal effects to avoid reverse causality and confounding, and to ensure the accuracy and robustness of the results. Thirdly, additional GWAS data used for replicate analysis and meta-analysis were further validated, supporting the reliability of causal inference of certain metabolites with PCOS. In summary, genetic associations identified at the level of genetic variation will provide a metabolomics perspective for screening and identifying significantly altered metabolites that influence PCOS pathogenesis. This is expected to provide new markers for predicting those at increased risk of PCOS. Besides, Studies have confirmed that metabolic interventions by diet, exercise and lipid-lowering drugs are important treatments for PCOS, which can change the blood metabolites of PCOS (47). Our findings may provide preliminary evidence for the mechanistic targets of metabolic interventions in the treatment of PCOS, providing valuable insights for the design of future clinical studies.

However, some limitations need to be considered. Firstly, Due to the insufficient IVs, we relaxed the p-value threshold (*P*<1×10−5) for screening SNPs related to lipid metabolites. This is considered reasonable threshold in some studies (48, 49). This would lead to weak IVs bias that should be considered, although the F-statistics > 10. Secondly, although we used sensitivity analyses to exclude horizontal pleiotropy, we don’t have the strict screening and eliminate potential confounding factors related IVs. Besides, our study may be biased by associations of unknown confounders with IVs. Thirdly, age and weight are recognized as significant factors affecting the onset and treatment of PCOS. However, individual patient details were not available from publicly available GWAS databases, so subgroup analyses for age, weight, and underlying diseases could not be performed in the MR study. Differences in lipid metabolites in different phenotypes of PCOS may cause an overall causal effect bias. Finally, exposure and outcome GWAS data consisted of individuals of European ancestry, which while reducing ethnic heterogeneity, but restrict the applicability of our findings to other ethnic groups. This demographic bias may bias the findings and findings should be validated in different ethnic groups.

## 5 Conclusion

Our study confirmed the robustness of the causal effect of hexadecanedioate and dihomo-linolenate on PCOS risk at the genetic level. Blood lipid metabolites may potentially regulate the progression of PCOS by interfering with the biosynthesis of unsaturated fatty acids and primary bile acid biosynthesis pathways. The findings of MR provide a reference direction for the study of the pathogenesis of PCOS mediated by metabolomics. Clinical and mechanism studies in the future are needed to confirm the significance of the identified metabolites as clinical biomarkers for modulating PCOS risk and their potential target roles in treatment.

## Data availability statement

The datasets presented in this study can be found in online repositories. The names of the repository/repositories and accession number(s) can be found in the article/**Supplementary Material**.

## Ethics statement

Ethical approval was not required for the studies involving humans in accordance with the local legislation and institutional requirements because this study applied publicly accessible GWAS data. Informed consent for studies included in the analyses was provided in the original publications.

## Author contributions

XW: Methodology, Writing – original draft. HH: Conceptualization, Data curation, Writing – original draft. XS: Writing – review & editing. XN: Writing – review & editing. RZ: Writing – review & editing. JJ: Funding acquisition, Writing – original draft. HZ: Funding acquisition, Supervision, Writing – original draft.
