# Supplementary material for: Genetic insights of blood lipid metabolites on polycystic ovary syndrome risk: a bidirectional two-sample Mendelian randomization study
Source: Front Endocrinol (Lausanne). 2024 Jul 9;15:1391826. doi: 10.3389/fendo.2024.1391826 (PMC11263216; doi:10.3389/fendo.2024.1391826)
Supplement: Supplementary file 2 [file DataSheet_1.docx]

Supplementary Material

**Supplementary Figure 1** **Scatter plots of causal estimates of exposure (Specific lipid metabolites) on outcome (PCOS).**

The slope of each line corresponding to the estimated MR effect in different models, including the conventional IVW, Weighted median, MR-Egger, Simple mode, and Weighted mode. The lines implying positive correlations moved diagonally upward from left to right, indicating a facilitative effect of lipid metabolites on PCOS. The lines implying negative correlations move diagonally downward from left to right, indicating the inhibitory effect of lipid metabolites on PCOS. The horizontal and vertical lines indicated each correlation’s 95% confidence interval.(a):*Hexanoylcamitine;* (b): *3-dehydrocarnitine*; (c): *1-arachidonoylglycerophosphoethanolamine*; (d): *2-tetradecenoyl carnitine*; (e): *Hexadecanedioate*; (f): *Dihomo-linolenate*; (g): *7-alpha-hydroxy-3-oxo-4-cholestenoate*;


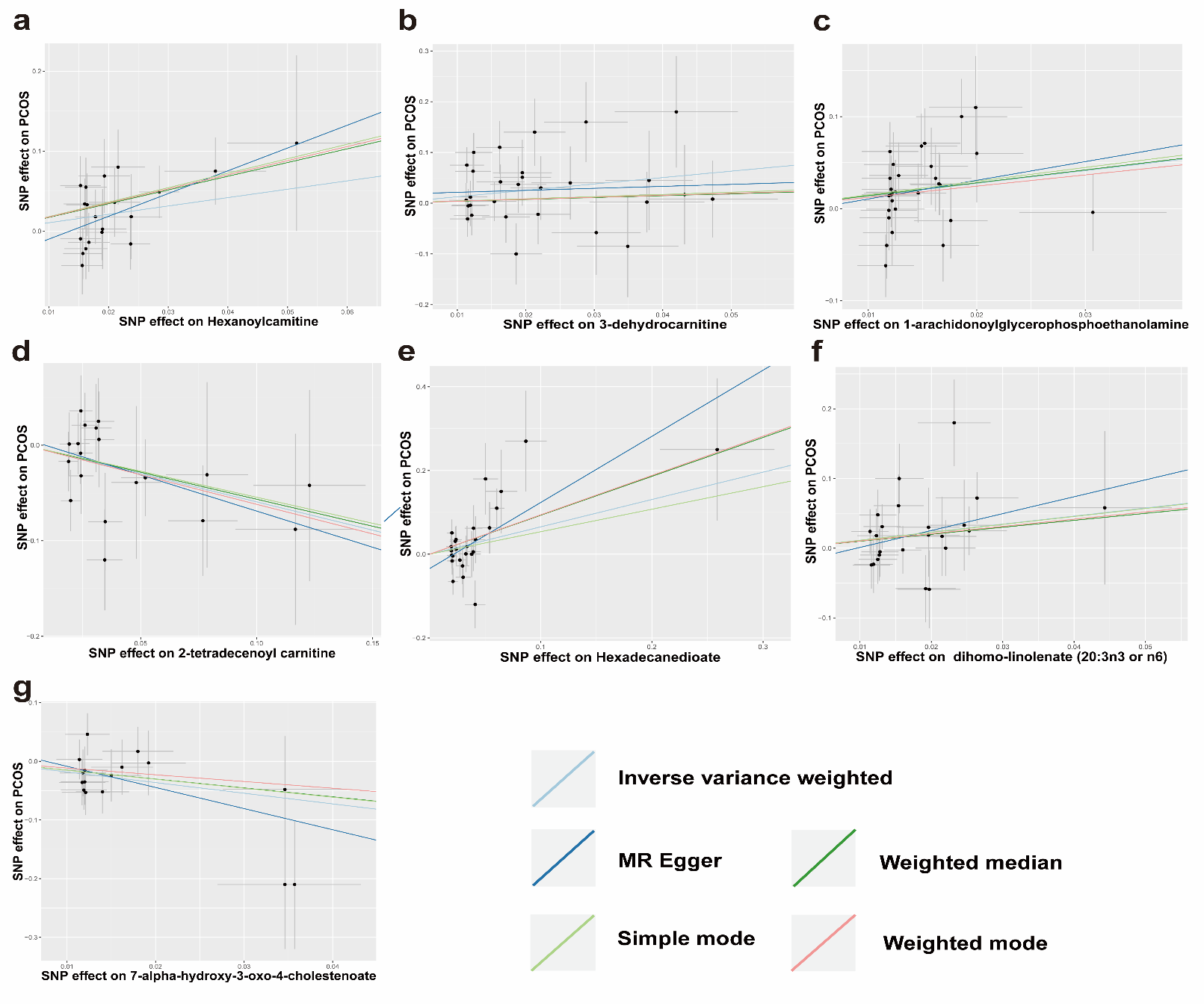


**Supplementary Figure 2**  **Funnel plots** **of causal estimates of exposure (Specific lipid metabolites) on outcome (PCOS).**

**
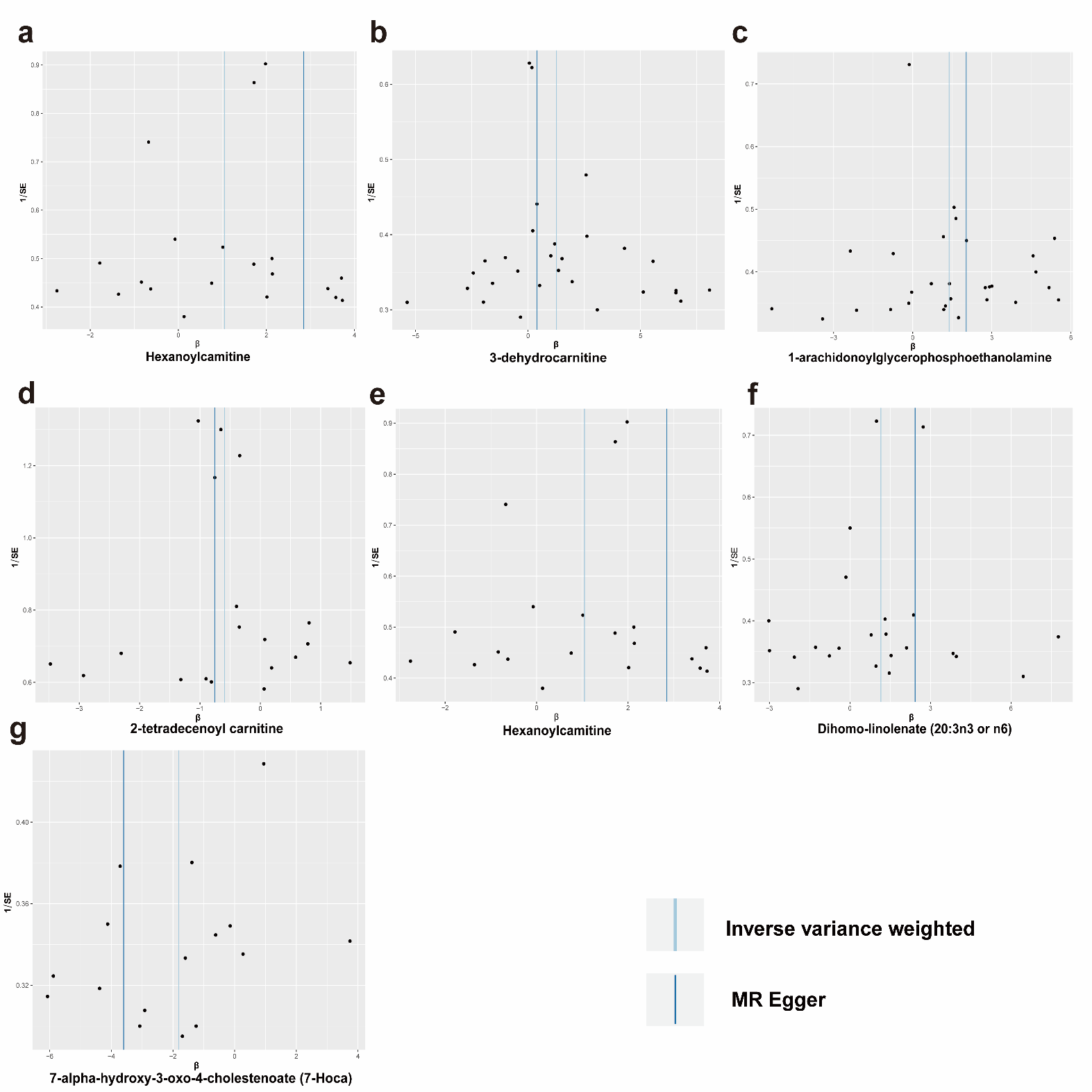
**IVW and MR-Egger show the approximate distribution of SNPs included in MR. The points on the left and right sides of the IVW line are roughly symmetrical, indicating the absence of outliers. (a):*Hexanoylcamitine*; (b): *3-dehydrocarnitine*; (c): *1-arachidonoylglycerophosphoethanolamine*; (d): 2-tetradecenoyl carnitine; (e): *Hexadecanedioate*; (f): Dihomo-linolenate; (g): *7-alpha-hydroxy-3-oxo-4-cholestenoate*;

**Supplementary Figure 3** **
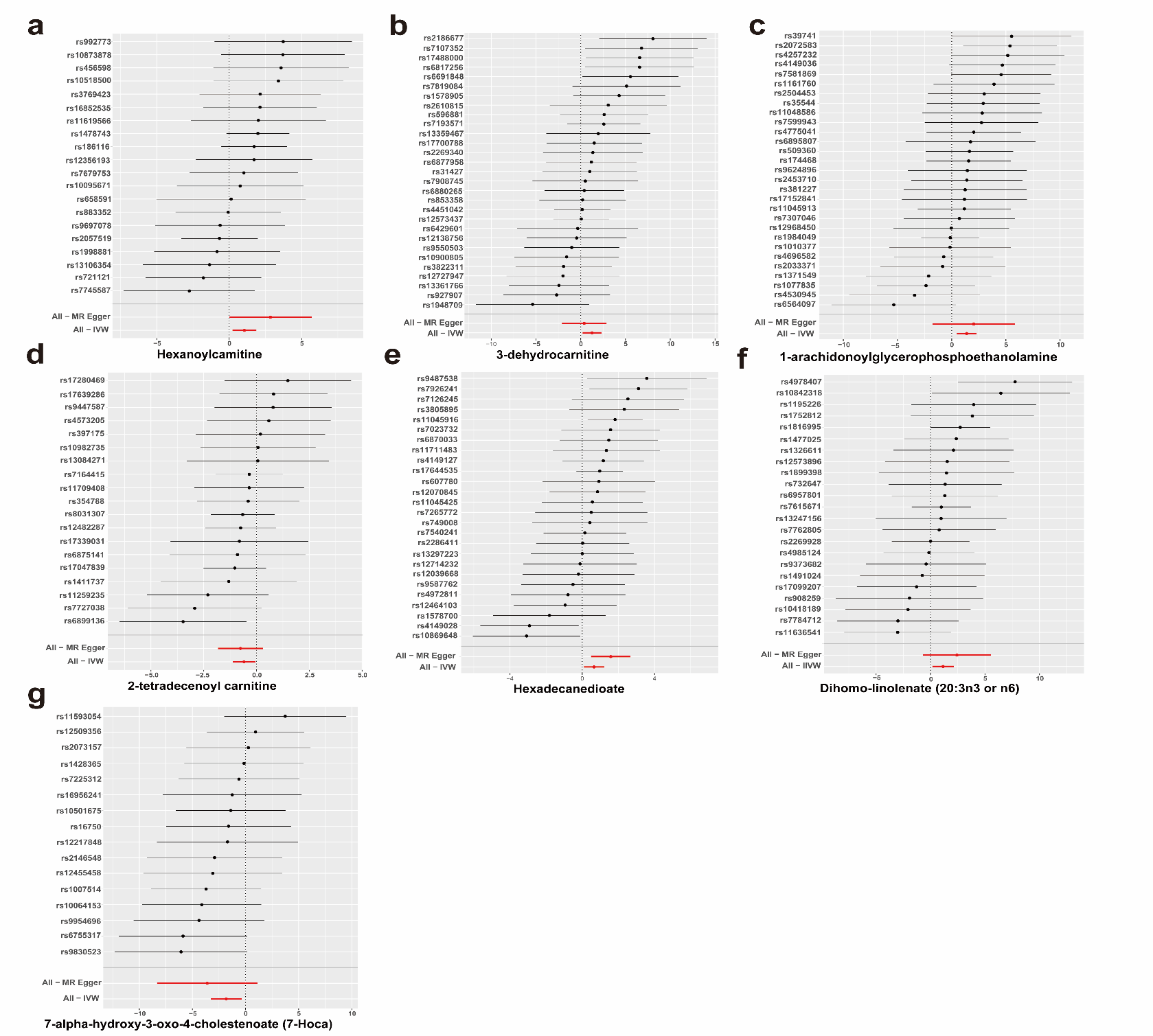
 Forest plots of causal estimates of exposure (Specific lipid metabolites) on outcome (PCOS).**

MR Effect of individual SNPs in different lipid metabolites and PCOS. The dots represent the estimates, and the bars represent the 95% confidence intervals of estimates. (a): *Hexanoylcamitine*; (b): *3-dehydrocarnitine*; (c): *1-arachidonoylglycerophosphoethanolamine*; (d): *2-tetradecenoyl carnitine*; (e): *Hexadecanedioate*; (f): *Dihomo-linolenate*; (g): *7-alpha-hydroxy-3-oxo-4-cholestenoate*;
